# Post-operative infection following ankle fracture surgery: a current concepts review

**DOI:** 10.1007/s00068-025-02837-3

**Published:** 2025-03-29

**Authors:** Jasper Tausendfreund, Jens Halm, Erik Tanis, Michael Swords, Tim Schepers

**Affiliations:** 1 Trauma Unit, Amsterdam University Medical Center, Amsterdam Movement Sciences, Meibergdreef 9, 1105 AZ Amsterdam, The Netherlands; 2 Trauma Unit, Noordwest Hospital Group, Wilhelminalaan 12, 1815 JD Alkmaar, The Netherlands; 3 Michigan Orthopedic Center, University of Michigan Health-Sparrow, 1215 Michigan Ave, Lansing, MI USA

**Keywords:** Ankle, Surgical site infection, Fracture-related infection, Risk factors, Open reduction internal fixation

## Abstract

**Purpose:**

The most common early complication of operative treatment of ankle fractures is a surgical site infection (SSI) with an incidence rate varying between 1.5 and 16%, depending on various risk factors. A SSI has multiple disadvantages, including worse outcome and a socio-economic burden. The aim of this review is to provide an updated overview of the current concepts pertinent to SSI in ankle fractures.

**Methods:**

A descriptive literature review was performed to provide the overview.

**Results:**

Well known risk factors for SSI are higher age, diabetes, open fractures and fracture dislocation. Diagnostic testing for infection include laboratory results (CRP, white blood cell count, leucocyte count), radiological imaging methods (conventional imaging, CT-scan, MRI-scan, 3-phase bone scan, FDG-PET) and microbiological deep tissue sampling. Treatment options for SSI are varied and include fracture reduction, antibiotic therapy with intravenous and oral treatment, surgical debridement and irrigation, transposition flaps in case of soft tissue defects with implant exposure and arthrodesis in severe infection with septic arthritis. Multiple studies show worse outcome scores in patients who develop a SSI. Prevention is important to reduce the rate of SSI. Surgery within 24 h decreases the risk of complications, compared to surgery performed in a delayed fashion. Appropriate timing and dosing of preoperative antibiotic prophylaxis is necessary.

**Conclusion:**

This review described the most frequent risk factors, appropriate diagnostic testing methods, an oversight of treatment options, gives insight in the outcome and mentioned prevention measurements for SSI after ORIF in ankle fractures.

## Introduction

Ankle fractures are amongst the most common occurring orthopaedic traumatic injuries. The incidence of ankle fractures is estimated to be up to 107–187/100,000 person-years [1–7]. Several studies have shown a rise in incidence over the years [6, 8, 9].

Between 30 and 50% of ankle fractures are treated operatively, however percentages up to 70% have been reported [2, 9, 10].

The most common early complication of operative treatment of ankle fractures is surgical site infection (SSI). The rate of SSI varies significantly throughout the literature. In studies, not specifically investigating certain high-risk populations (e.g., elderly, diabetics), the rates of SSI following open reduction and internal fixation (ORIF) vary between 1.5 and 17% [11, 12]. Studies on deep infections reported incidences between 2.8 and 6.8% [13, 14].

The importance of reducing SSI is made clear in studies investigating the outcome in patients with a SSI after operative treatment of an ankle fracture. SSI leads to worse outcome [15–17]. In addition to the negative effects of an SSI for the patient in relation to functional outcome, SSIs carry a large socio-economic burden. Prevention of SSI is of paramount importance as patients with SSI may have a median of 3.1 up to 7.6 times higher hospital costs based on a five to ten-fold longer hospital stay, prolonged antibiotic treatment and additional surgical procedures [18].

This study aims to provide an overview on surgical site infection in patients after operative treatment of an ankle fracture.

## Methods

A wide literature search was performed to provide a descriptive literature review. Studies in English, German and Dutch were reviewed for information. A search was performed for studies on definitions and classifications, epidemiology, risk factors and predictors, diagnostics, treatment options, outcome and prevention.

## Definitions and classifications of infection

Definitions of infection varies between studies. Some studies define infection using the terms osteomyelitis and dehiscence [19], others studies categorize infection severity by dividing infection into cellulitis versus return to the operation room [20], or superficial/deep [15]. Other terms, such as wound edge necrosis and impaired or delayed wound healing are also used as descriptors [21].

In patients with infection after surgery, multiple terms are used. Most commonly used is SSI. Commonly used terms to describe infection in fracture related surgery include fracture related infection (FRI) and infection after fracture fixation (IAFF) [22, 23]. These varying terms have some differences in definition, but also contain a certain amount of overlap as well.

The CDC guideline provides an overview of the definition of SSI, superficial and deep incisional infection [24, 25]. FRI is often classified as early (< 2 weeks), delayed (3–10 weeks) or late (> 10 weeks) [26]. These time frames are expected to be related to different symptoms and biofilm formation [27]. Although recent recommendations are to establish a singular definition for FRI as there is no evidence for the relation of time frame to infection [23]. Govaert et al. [22], describe suggestive criteria for FRI as local redness, swelling, increased local temperature, fever (≥ 38.3 °C), persistent, increasing or new-onset wound drainage beyond the first few days postoperatively, radiological signs of FRI (osteomyelitis and/or soft tissue involvement), new-onset joint effusion, elevated serum inflammatory markers and confirmatory criteria as pus, fistula, sinus or wound breakdown.

A new classification for FRI was proposed by Pilskog et al. [28]. It is based on fracture characteristics, relevant systemic comorbidities of the patient, and impairment of soft tissue. The classification has five stages and focusses on disease elements which need treatment.

The aforementioned criteria were assessed in a cohort study which showed a 10% suspected infection rate. Of those with suspected infections, 73% met the confirmatory criteria and 27% the suggestive criteria. 39% of the patients who met the suggestive criteria had positive cultures. This diagnostic algorithm provides an approach for FRI [29].

In case of osteomyelitis, the Cierny–Mader classification provides a staging system that is based on the extensiveness of the bone involvement and the subjective physiological class (immune status) of the host with its characteristics and affecting comorbidities [30]. The BACH classification was developed for long bone osteomyelitis and was assessed to be an accurate system. It contains bone involvement, antimicrobial resistance patterns of the causative pathogens as well as soft tissue coverage, and host status [31, 32].

## Epidemiology of infection after surgically treated ankle fractures

Studies on the incidence of SSI after ORIF of ankle fracture vary between large national database studies, prospective cohort studies and systematic reviews. Patients with diabetes, alcohol abuse and the general population were all compared [33–35]. Table 1 summarizes the rate of ankle fracture SSI reported in the literature, including incidence percentages. SSI rates vary from 1.4 to 17% for SSI in the general population.

**Table 1:** Rates of SSI in operatively treated ankle fractures

Study	Number of patients	Study type	SSI (%)^a^
Basques et al. [36]	4412	NSQIP database	1.75
Belmont et al. [37]	3328	NSQIP database	3.4
Bergström et al. [38]	480	Retrospective	10.2
Blotter et al. [39]	67	Retrospective Diabetes versus control	33 versus 4
Cammas et al. [40]	433	Retrospective	6^b^
Carragee et al. [41]	121	Retrospective	13.9
Chien et al. [42]	161	Retrospective	17
Cooke et al. [43]	1003	Retrospective	15
Costigan et al. [44]	84	Retrospective Diabetes	13
Dodd et al. [11]	6865	NSQIP database	1.5
Flynn et al. [45]	98	Diabetes versus control	10.5 versus 9
Han et al. [46]	613	Retrospective	5.2
Hoiness and Stromsoe [47]	118	Retrospective	5.9
Hoiness et al. [48]	154	Prospective	5.8
Jones et al. [49]	82	Retrospective Diabetes versus control	14.3 versus 2.4
Kang et al. [50]	41,071	National cohort	2.1
Keene et al. [51]	309	Prospective	2.9
Kelly et al. [52]	122	Prospective	9.8
Korim et al. [15]	717	Retrospective	4.1
Lachman et al. [20]	1442	Retrospective	4.9
Lillmars and Meister [33]	356	Meta-analysis Diabetic versus control	25 versus 6
Lindsjo [53]	321	Prospective	1.8
Liu et al. [54]	1532	Retrospective	2.9
Lynde et al. [19]	216	Retrospective	10.6
Macera et al. [21]	378	Retrospective	4.7
Mak et al. [55]	116	Retrospective	8.6
Meng et al. [13]	2617	Retrospective	2.8^b^
Meng et al. [56]	1201	Retrospective	2.1
Miller et al. [57]	478	Retrospective	4.2
Nasell et al. [58]	906	Retrospective	12.5
Naumann et al. [17]	567	Retrospective	5.1
Olsen et al. [59]	1043	Retrospective	14
Ovaska et al. [60]	1923	Retrospective	6.8^b^
Ovaska et al. [12]	137	Retrospective	17^b^
Penning et al. [61]	447	Retrospective	11.2
Pilskog et al. [62]	1004	Retrospective	9
Rascoe et al. [63]	776	Retrospective	7.2
Richardson et al. [64]	3687	Database	10.7
Richter et al. [65]	647	Retrospective	4.5^b^
Sato et al. [66]	1201	Retrospective	5.7
Schepers et al. [67]	205	Retrospective	7.8
Shao et al. [34]	8103	Meta-analysis	7.2
Smeeing et al. [68]	989	Retrospective	7.4
SooHoo et al. [69]	57,183	Database	1.44
Still and Atwoord [70]	41	Retrospective	2.4
Sun et al. [71]	1247	Retrospective	3.7
Sun et al. [72]	1510	Retrospective	4.4^c^
Tan et al. [73]	45	Complicated versus uncomplicated diabetes	50 versus 6
Thangarajah et al. [74]	50	Retrospective	24
Tonnesen et al. [35]	180	Alcoholics versus controls	16.7 versus 4.4
Tunturi et al. [75]	124	Retrospective	7.3
White et al. [76]	100	Nail versus plate	0 versus 16
Zaghloul et al. [77]	186	Retrospective	16

^a^SSI (surgical site infection) % in ankle fractures (not including dehiscence and wound edge necrosis)

^b^Deep infections reported only

^c^During hospitalisation

Certain subsets of patients were associated with even higher incidences. Alcohol abusers showed incidence of 16.7% compared to 4.4% in the general population of that cohort [78]. While patients with uncomplicated diabetes were comparable to the general population [73], patients with poorly controlled diabetes or diabetic complications were found to have an SSI incidence up to 50%. This group included patients with neuropathy, retinopathy, renal impairment, cerebral or peripheral vascular disease, ischemic heart disease or end organ dysfunction. Patients with open fractures were found to have a deep SSI incidence of 17% [12].

These studies had different outcome variables and definitions for complications and infections. Some did not distinguish between superficial and deep infection, others only included deep infections. In Table 1, we excluded studies that did not describe specific percentages of SSI, but only described wound complications. The large database studies showed substantially lower incidence of SSI, which might be caused by underreporting and they usually only reported the 30-day morbidity [11, 36, 37].

## Risks factors and predictors of infection

Risk factors for SSI are widely studied and vary in the literature. In Table 2, we included the most frequently reported significant uni- and multivariate risk factors for SSI after ORIF of ankle fractures. Risk factors divided in patient related, injury related and surgery related characteristics.

**Table 2:** Case series: factors related to increased wound infection in univariate and multivariate analysis

*Patient related characteristics*	
Age	Andrés-Peiró et al. [79], Basques et al. [36], Belmont et al. [37], Bergström et al. [38], Dodd et al. [11], Han et al. [46], Kelly et al. [52], Lynde et al. [19], Meng et al. [13], Miller et al. [57], Nasell et al. [58], Pilskog et al. [62], Richter et al. [65], Smeeing et al. [68], SooHoo et al. [69], Sun et al. [72], Zaghloul et al. [77]
Alcohol	Hoiness et al. [48], Meng et al. [13], Olsen et al. [59], Ovaska et al. [60], Shao et al. [34], Tonnesen et al. [35]
ASA-score	Belmont et al. [37], Rascoe et al. [63], Richter et al. [65], Shao et al. [34], Smeeing et al. [68]
Chronic heart disease	Meng et al. [13], Pilskog et al. [62], Sato et al. [66], Shao et al. [34]
Dependent functional status	Backes et al. [80], Basques et al. [36], Juto et al. [4]
Diabetes	Basques et al. [36], Blotter et al. [39], Cooke et al. [43], De Boer et al. [9], Flynn et al. [45], Jensen et al. [2], Korim et al. [15], Lynde et al. [19], Miller et al. [57], Nasell et al. [58], Ovaska et al. [60], Penning et al. [61], Rascoe et al. [63], Richter et al. [65], Shao et al. [34], SooHoo et al. [69], Zaghloul et al. [77]
Gender (female)	Kelly et al. [52], Liu et al. [54]
Gender (male)	Cooke et al. [43], Richardson et al. [64]
History of allergy	Shao et al. [34], Sun et al. [72]
Malnutrition	Meng et al. [13]
Neuropathy	Costigan et al. [44], Flynn et al. [45], Lillmars and Meister [33], Miller et al. [57], Zaghloul et al. [77]
Non-compliance	Miller et al. [57]
Nursing home	Korim et al. [15]
Obesity	Bengner et al. [8], Olsen et al. [59], Riedel et al. [81], Richardson et al. [64], Shao et al. [34], Sun et al. [72]
Peripheral vascular disease	Belmont et al. [37], Costigan et al. [44], Flynn et al. [45], Lillmars and Meister [33], Pilskog et al. [62], Richardson et al. [64], Richter et al. [65], Soohoo et al. [69], Zaghloul et al. [77]
Renal disease	Rascoe et al. [63]
Smoking	Andrés-Peiró et al. [79], Belmont et al. [37], Cooke et al. [43], Meng et al. [13], Nasell et al. [58], Ovaska et al. [14], Ovaska et al. [60], Pilskog et al. [62], Richardson et al. [64], Riedel et al. [81], Sato et al. [66], Thangarajah et al. [74], Zaghloul et al. [77]
Specific medication	Cooke et al. [43], Miller et al. [57]
*Injury related characteristics*	
Bi- trimalleolar	Basques et al. [36], Daly et al. [1], Hoiness et al. [48], Hoiness and Stromsoe [47], Nasell et al. [58], Richter et al. [65], Sato et al. [66], Thangarajah et al. [74]
Fracture-dislocation	Ovaska et al. [60], Shao et al. [34]
Heelpad edema	Riedel et al. [81]
High energy trauma	Hoiness et al. [48], Shao et al. [34], Sun et al. [72]
Initial external fixation	Smeeing et al. [68]
Non-clean wound	Belmont et al. [37], Sun et al. [72]
Open fracture	Belmont et al. [37], Bergström et al. [38], Carragee et al. [41], Han et al. [46], Meng et al. [13], Miller et al. [57], Nasell et al. [58], Ovaska et al. [60], Penning et al. [61], Rascoe et al. [63], Shao et al. [34], Smeeing et al. [68], Sun et al. [72]
Weber-C	Korim et al. [15], Ovaska et al. [14]
*Surgery related characteristics*	
Delayed surgery	Carragee et al. [41], Hoiness and Stromsoe [82], Schepers et al. [16]
Drain usage	Kelly et al. [52]
Early motion	Keene et al. [83], Thomas et al. [84]
Locking plates	Lynde et al. [19], Schepers et al. [67]
Malreduction	Nasell et al. [58], Ovaska et al. [60]
Peri-operative pyrexia	Kelly et al. [52]
Prolonged surgery	Andrés-Peiró et al. [79], Gowd et al. [85], Meng et al. [13], Ovaska et al. [14], Ovaska et al. [60], Sun et al. [72]
Timing of antibiotics	Ovaska et al. [60]

*ASA* American Society of Anaesthesiologists

One of the frequently described risk factors is age, although different cut-off points for higher age were used, most of them starting at 60 years, nonetheless it seems to be an important risk factor. Other patient related risk factors that were described include obesity, peripheral vascular disease, smoking, alcohol abuse and diabetes.

Diabetes is a well-known risk factor for SSI. A systematic review of 40 studies showed that patients with diabetes had higher complication rates then the general population. Complication rates are even higher in insulin dependent diabetes, poorly controlled diabetics, and in patients with diabetes related complications. Non-operative treatment and external fixation for patients with diabetes are also associated with higher complication rates [86].

Open fracture is an important injury related risk factor. As mentioned previously, an open fracture leads to an increased incidence of deep SSI [12]. Literature review on open ankle fractures has shown no consensus on the timing of surgery. Gold standard for initial management of open ankle fractures is wound irrigation and debridement [87]. Immediate ORIF resulted in good function outcome, less joint stiffness and shorter hospital stay compared to delayed or conservative treatment [88]. If soft tissue coverage of the implant is not possible, two-stage treatment with a temporary external fixator should be considered [88].

Bi- and trimalleolar fracture pattern and fracture dislocation are frequently described as risk factors for SSI [36, 66], this might be related to the increased risk of soft tissue injury and open fractures. Prolonged surgery has also been shown to be a significant surgery related risk factor [13, 14, 60, 72, 79]. A prediction algorithm for risk at SSI could be performed for individual patients. It includes a scoring system that estimate the risk depending on the comorbidities of the patient. In patients above 65 years old, the modified Charlson comorbidity index serves as a predictor for complications. Although further research for a validated scoring system is still necessary [77].

## Diagnostics

In case of suspected SSI, the clinical findings need to be standardized and well defined.

### Laboratory tests

The most used biomarker for infection is C-reactive protein (CRP). There is no evidence for a cutoff value of CRP in SSI after ORIF of ankle fractures, although there are some studies for orthopedic and (non-ankle) fracture surgery, that suggest the expected peak of CRP after prosthetic surgery is at post-operative day 2–3 [89, 90]. After that day CRP should decline to normal range in about 2–3 weeks [89]. In fracture surgery a lower peak CRP was seen, but a similar decline pattern. Open fractures and extensive trauma mechanism showed a higher CRP value. For ankle fractures the mean peak CRP was around 34-39 mg/L and showed CRP as a predictor for infection in fracture surgery has showed a sensitivity 60–100%, specificity of 65–98.4%, negative predictive value 17% and positive predictive value 98% [89]. A cut off value of 96 mg/L with a sensitivity 92% and specificity 93% was seen after post-operative day 4 [90]. This value may aid in early detection if other infections are ruled out. A single CRP value is not considered useful, a baseline CRP or day 2 CRP is required [90]. A significant difference in CRP between postoperative day 3 and 7 can be used as a reliable predictor for infection [91].

Other laboratory results which were evaluated as diagnostic marker in spine surgery were white blood cell (WBC) count, these show a maximum value on postoperative day 1–3 and should decline to normal within 4–6 days [92]. Cutoff values for increased white blood count are well established however the differential diagnosis of leukocytosis is vast [93].

Govaert et al. [22], reviewed the diagnostic value of CRP, lymphocyte count (LC) and erythrocyte sedimentation rate (ESR). They suggest that all the three markers individually have limited diagnostic value and are not applicable to rule out chronic or late-onset FRI and they should only be used as a suggestive criteria for FRI.

### Radiological imaging

There are a number of indications for radiological imaging in case of suspected SSI. (1) Acquire additional information on the absence or presence of SSI, (2) to get details on the disease extension and anatomical changes (abscess, sinus tract or sequester), (3) assess implant stability and fracture consolidation. Commonly used methods include conventional radiograph, computed tomography (CT), magnetic imaging resonance (MRI), 3-phase bone scan (BS), fluorodeoxyglucose positron emission tomography (FDG-PET), and white blood cell (WBC) scintigraphy. Table 3 shows a modified table published by Govaert et al. [22], with the sensitivity, specificity, advantages, and disadvantages of the different techniques [22]. A useful modality of the FDG-PET-scan is the maximum standardized uptake value (SUVmax). It has shown to be promising in discriminating between septic and aseptic delayed union with a sensitivity of 65%, specificity of 77% and diagnostic accuracy of 70% [94, 95].

**Table 3:** Imaging methods. Modified table from Govaert et al. [22]

Imaging technique	Advantages	Disadvantages	Sensitivity	Specificity
Conventional radiograph	Easily available, cheap, low radiation exposure	No clear details	–	–
CT-scan	Better details: sequestra/bone cavities	Higher radiation exposure	47%	60%
MRI-scan	Details on soft tissue pathology/bony changes/sequestra/sinus tracts/abscesses/cloacae	Metal implants result in scattering	82–100%	43–60%
BS	Better anatomic details	Very low specificity	89–100%	0–10%
WBC scintigraphy + SPECT	No influence of recent surgery	Time consuming (2 scans), Less accurate for axial skeleton	79–100%	89–97%
FDG-PET scan	High spatial resolution, better in quantification	Less accurate than WBC scintigraphy, not useful within 1 month postoperatively	65–94%	76–100%

*CT* computed tomography, *MRI* magnetic resonance imaging, *BS* bone scintigraphy, *WBC* white blood cell, *SPECT* single photon emission computed tomography, *FDG-PET* fluorodeoxyglucose-Positron emission tomography

### Microorganism sampling

A study of prosthetic joint infections (PJI) by Atkins et al. [96], advised to obtain at least five deep tissue samples during surgical debridement. The study showed that isolation of microorganism in three or more independent samples had a sensitivity of 65% and specificity of 99.6%.

## Treatment

### Fracture reduction

Immediate reduction of fracture dislocation at the trauma site or emergency department are often performed to reduce pain, prevent ischemia and nerve damage [97]. The quality of the preoperative closed reduction has no association with the incidence of post-operative wound complications. Thus, multiple attempts to achieve perfect reduction are unnecessary [42].

Further research on the association between preoperative reduction and SSI rate may provide better evidence for recommendations. Delay in reduction may result in soft tissue compromise such as skin blisters and excess swelling resulting in a potential increased risk of SSI from surgery performed through traumatized tissues. Delay of surgical management may potentially increase the difficulty of surgical reduction due to early fracture fibrosis.

### Antibiotics

Treatment with antibiotics is often initiated in case of SSI. In complex orthopaedic infections, oral administration of antibiotics was noninferior to intravenous therapy in the first 6 weeks [98]. Expert group recommendations exist for the duration of antibiotic therapy. Treatment duration of 12 weeks is generally recommended, starting with intravenous administration, until soft tissue infection has settled and full antibiotic susceptibility patterns are available. This could be as short as one week of intravenous treatment, followed by 11 weeks oral treatment. It is advised to use biofilm-active therapy if possible. If the treatment approach is suppressive and not eradicative, antibiotics should be continued until 1 or 2 weeks after fracture consolidation and implant removal [99].

Current guidelines for antibiotic treatment of bone and prosthetic joint infections recommends empirical therapy based on the suspected causative pathogens. In DAIR procedures (Debridement, Antibiotics, Implant Retention) for early infections or one-stage revision surgery for late infections the recommended antibiotics are vancomycin and ceftazidime [100]. Guidelines often differ per country and different antibiotics are recommended in case of specific suspected bacteria. Antibiotics should be based on sensitivity data when possible.

In case of suspicion of early infection with Staphylococcus Aureus or Coagulase-negative Staphylococci (CNS), first choice of therapy is flucloxacillin with rifampicin. In case of flucloxacillin resistance, vancomycin with rifampicin is recommended. Initial coverage for Enterobacteres is accomplished with ceftriaxone [100].

### Surgical debridement

In the literature, many studies suggest that surgical debridement of infected tissue and irrigation is the most effective treatment [32]. There is no standard for which solution should be used for irrigation, nor for the technique or quantity of fluid [101]. Pulsatile lavage has no additional value and could even be a risk factor for wound necrosis [102]. To our knowledge, the effect of surgical tissue debridement has never been studied. DAIR procedures had a lower eradication rate in patients with periprosthetic ankle infection (58.8%) compared to permanent antibiotics spacers (91.7%), 2-stage surgery (84.4%) and arthrodesis (79.4%) in prosthetic ankle infections [103]. Even though it is common practice, results of DAIR procedures in ORIF for ankle fractures has never been studied. Further studies on the effectiveness of the DAIR procedure may be necessary to expand our knowledge.

### Implant retention

Implants with adequate position and uncompromised stability and soft tissue coverage should not be removed [104, 105]. In patients with Enterobacter complex infections, implant salvage is considered more challenging, possibly because of the strong colonization of Enterobacter on medical devices [106].

Berkes et al. [107], showed that in postoperative SSI, 70.7% of patients had bony union and 29.3% non-union. Implant removal due to infection occurred in 21.1% in all cases. In the patients with bony union, 26 of the 121 required implant removal due to recurrence of infection. In the failure of treatment group, 27 patients had implant removal prior to union or non-union leading to revision or fusion. In four cases, implant removal was performed during the first debridement. Non-significant risk factors for failure were ankle surgery, smoking and pseudomonas infections. In cases of multiple risk factors, early implant removal may be considered [107].

### Transposition flaps

In case of soft tissue defects with exposure of implants following deep SSI, one of the surgical treatment options is flap reconstruction after appropriate debridement of infected tissues. Flaps commonly used for infection after ankle surgery include a distally based peroneus brevis muscle flap with split skin graft, sural artery perforator flap, microvascular free flap, perforator propeller flap or direct cutaneous flap. Flap reconstruction leads to retention of implants in 53% of the cases, but has downsides such as additional surgery, donor site morbidity, and footwear limitations [108].

### Arthrodesis

In cases of severe infection, cartilage may be damaged due to development of septic arthritis. Consequently, arthrodesis might be necessary to contain the infection and treat painful arthritis. Several studies with different approaches assessed the value of arthrodesis [109–111]. Surgical stabilization decreases trauma to both the soft tissues and the microcirculation. Stability leads to less edema and a decrease in infection rate. Although high complication rates of up to 44% are reported, union rates in the septic period were 76–86% and 80–95% in aseptic period [109].

Often the treatment included resection of infected tissue and compression with a hybrid frame, Ilizarov external fixator, compression screws (with anterior plate), for prolonged period of time in combination with antibiotic therapy [111–113].

Other treatment techniques were the Crawford-Adams and the Meary technique, with a success rate of respectively 91% and 87.5% [114].

Another option is debridement and sequestrectomy, followed by filling the void with gentamycin beads and placement of an external fixator for stability, followed by delayed bone graft after the acute infectious period has been appropriately managed. This strategy led to stable arthrodesis in 92.7% and a AOFAS score in follow up of 63.7 [110, 115].

### Cement spacer/PMMA

Polymethyl methacrylate antibiotic beads or spacers can be applied after bone and soft tissue debridement to deliver local antibiotics, add stability after bone resection, and serves to fill dead space to prevent room for hematoma, which be a medium for bacterial growth [109]. At removal of the PMMA, 90.4% of the patients had no bacterial growth [116]. The cement spacer enables patients to be mobile with less discomfort. In low demand patients with comorbidities this may be a viable definitive solution [117]. In low demand patients with foot infections, retainment, or exchange of the spacer for periods up to respectively 76 and 111 months, was successful for two-third of the population [118].

## Outcome

In 2013, Schepers et al. [16], compared 87 operatively treated ankle fractures without a SSI versus 14 with a SSI and saw a significant lower outcome on three different outcome scores after an average follow-up of 43 months. Later, these results were confirmed by Korim et al. [15], in a series of 706 patients (29 of which had a SSI), showing a significantly lower score on the Olerud Molander Ankle Score (OMAS). A third, more recent, study by Naumann et al. [17], showed significantly lower OMAS and Lower Extremity Functional Scale (LEFS) in 567 patients of which 29 experienced a SSI (Table 4).

**Table 4:** Outcome scores after ORIF for ankle fractures

Study	Number of patients	OMAS for no-SSI [IQR]	OMAS for SSI [IQR]	p value
Schepers et al.^a^ [16]	101	90 [80–100]	80 [66–90]	P = 0.016
Naumann et al. [17]	462	80 [60–100]	Superficial 75 [50–90] Deep 64 [62–76]	P = 0.001
Korim et al. [15]	52	90 [80–95]	60 [46–69]	p < 0.001

*ORIF* open reduction internal fixation, *OMAS* Olerud-Molander Ankle Score, *SSI* surgical site infection, *IQR* interquartile range

^a^Any wound complication, edge necrosis, wound dehiscence, superficial infection, and deep infection (osteomyelitis)

Independent prediction factors for worse outcome in foot function index (FFI) and Short Musculoskeletal Function Assessment (SMFA) are obesity, female gender, tobacco/alcohol use, secondary procedures and multiple additional injuries [119]. Other independent risk factors for treatment failure were postoperative malreduction, implant removal in non-consolidated fractures and the need for two or more debridements [60].

## Prevention

### Blood glucose regulation

Blood glucose is an indicator for a higher risk of complications. Patients with and without complications were compared and a significantly higher Hemoglobin A1c was seen in the group with complications. An increase of 1% in HbA1c increased the odds of complications by 5%. Patients with neuropathy had 1.78 times increased risk of complications and those who had 2 or 3 diabetic comorbidities had even higher risks of 3.08 times [120]. Therefore, blood glucose optimization is highly recommended peri operatively.

### Immobilization

After ankle fracture surgery, patients are often treated with restricted weight-bearing for a period of 6 weeks. However, early weight-bearing is still an active study topic [121].

Early weight bearing is associated with improved ROM, and significantly better OMAS and SF-36 scores after 6 weeks. No differences in wound complications and infections were seen between early and late weight bearing [122]. Smeeing et al. [123], reported a higher OMAS score for the unprotected weight bearing group, but only after 6 weeks. Other follow-up points showed no significant differences in quality of life nor complications. Therefore, early weight bearing could be assessed as safe. A systematic review by Thomas et al. [84], showed that early motion is associated with quicker return to work and improved range of motion at 12 weeks, although cast immobilization had significantly less risk of wound infection. This makes it difficult to conclude which treatment is better. A suggestion might be that young patients may benefit from early motion and patients with risk factors may benefit from cast immobilization [84]. In addition, a recent randomized controlled trial found early weight-bearing to be clinically non-inferior, with better OMAS scores [121]. Unfortunately, 30 percent of patients were not compliant to the treatment prescribed.

### Timing of surgery

Surgery for closed ankle fractures after 24 h had an increase in complications [41]. Schepers et al. [16], assessed the timing of surgery and found no wound complications in the patients treated within one day, but 11% wound complications in the delayed group (surgery after 1 day). A systematic review of surgically treated fractures showed similar results with 3.6% of complications in the group treated early and 12.9% in the late group [16].

### Antibiotic prophylaxis

Administration of antibiotic prophylaxis should be performed within 120 min of the incision. Earlier than 120 min or after incision is associated with higher risk of SSI [124]. Fluoroquinolones and vancomycin have a long infusion time and should be started 120 min before incision. Infusions of cephalosporin, such as cefazolin, should be started within 60 min before incision [125]. No benefits were found for prolonged antibiotic prophylaxis postoperatively, when following best practice standards [126]. Application of topical vancomycin powder as prevention for SSI in fracture surgery of the foot and ankle of diabetic patients decreased the rate of deep infections by 80%, but there was no significant difference in occurrence of superficial SSI [127]. However, recent literature suggests the roll of vancomycin powder is still controversial. Vancomycin powder is used more commonly in open fractures [128].

### Soft tissue coverage

Immediate soft tissue coverage provides no significant differences in the frequencies of hardware removal, secondary plastic surgery procedures, ultimate failure and malunion, compared to patients who were referred to a plastic surgeon post-operatively [129].

## Conclusion

SSI after ORIF for ankle fractures is a common adverse event with an incidence of 1.4–17%. Multiple terms and definitions are employed in the literature. The CDC guideline and Govaert et al. [22], provide useful definitions of SSI and FRI for daily practice. Extensive literature is available about risk factors for SSI. The most frequently described factors include diabetes with complications, open fractures, age, obesity, peripheral vascular disease, smoking and alcohol abuse. Further research on comorbidity scoring systems, such as the modified Charlson comorbidities index, is still necessary. Diagnostic tools available include laboratory tests (CRP, WBC and LC), radiological imaging methods (conventional radiograph, CT, MRI, BS, WBC-scintigraphy and FDG-PET), and tissue sampling (≥ 5 deep samples). Treatment consists of fracture reduction, antibiotics, surgical debridement and irrigation. Antibiotics during one week intravenously followed by 11 weeks oral therapy, and in some cases suppressive therapy until implant removal. In early infections empirical treatment of *S. Aureus* or CNS with flucloxacilin and rifampicin. If there is concern for *Enterobacter* species, ceftriaxone is recommended. Patients with SSI showed significantly worse outcome in follow-up in multiple scoring systems (e.g. OMAS, LFES). For future perspectives, prevention is preferable over treatment. Patient selection for surgery using risk factors in assessment, and peri operative glucose management and optimization in diabetics could decrease complications. A 1% decrease in HbA1c has a 5% lower odds of surgical complications. If possible, surgery within 24 h leads to less complications. Antibiotic prophylaxis should be administrated adequately according to local protocols with attention to timing, dose and antibiotic selection. Improved outcomes are related to early post-operative mobilization and might be applicable for young patients without risk factors, but cast immobilization showed less wound infections.

## Data Availability

No datasets were generated or analysed during the current study.
